# The Fungicide Chlorothalonil Is Nonlinearly Associated with Corticosterone Levels, Immunity, and Mortality in Amphibians

**DOI:** 10.1289/ehp.1002956

**Published:** 2011-04-04

**Authors:** Taegan A. McMahon, Neal T. Halstead, Steve Johnson, Thomas R. Raffel, John M. Romansic, Patrick W. Crumrine, Raoul K. Boughton, Lynn B. Martin, Jason R. Rohr

**Affiliations:** 1 Department of Integrative Biology, University of South Florida, Tampa, Florida, USA; 2 University of Florida Gulf Coast Research and Education Center, Wimauma, Florida, USA; 3 Department of Biological Sciences, Rowan University, Glassboro, New Jersey, USA; 4 Avian Ecology Program, Archbold Biological Station, Venus, Florida, USA

**Keywords:** disease, endocrine disruption, immunity, mortality, pesticide, toxicology

## Abstract

Background: Contaminants have been implicated in declines of amphibians, a taxon with vital systems similar to those of humans. However, many chemicals have not been thoroughly tested on amphibians or do not directly kill them.

Objective: Our goal in this study was to quantify amphibian responses to chlorothalonil, the most commonly used synthetic fungicide in the United States.

Methods: We reared *Rana sphenocephala* (southern leopard frog) and *Osteopilus septentrionalis* (Cuban treefrog) in outdoor mesocosms with or without 1 time (1×) and 2 times (2×) the expected environmental concentration (EEC) of chlorothalonil (~ 164 μg/L). We also conducted two dose–response experiments on *O. septentrionalis*, *Hyla squirella* (squirrel treefrog), *Hyla cinerea* (green treefrog), and *R. sphenocephala* and evaluated the effects of chlorothalonil on the stress hormone corticosterone.

Results: For both species in the mesocosm experiment, the 1× and 2× EEC treatments were associated with > 87% and 100% mortality, respectively. In the laboratory experiments, the approximate EEC caused 100% mortality of all species within 24 hr; 82 μg/L killed 100% of *R. sphenocephala*, and 0.0164 μg/L caused significant tadpole mortality of *R. sphenocephala* and *H. cinerea*. Three species  showed a nonmonotonic dose response, with low and high concentrations causing significantly greater mortality than did intermediate concentrations or control treatments. For *O. septentrionalis*, corticosterone exhibited a similar nonmonotonic dose response and chlorothalonil concentration was inversely associated with liver tissue and immune cell densities (< 16.4 μg/L).

Conclusions: Chlorothalonil killed nearly every amphibian at the approximate EEC; at concentrations to which humans are commonly exposed, it increased mortality and was associated with elevated corticosterone levels and changes in immune cells. Future studies should directly quantify the effects of chlorothalonil on amphibian populations and human health.

Amphibians are arguably the “poster child” of the present extinction crisis (Wake and Vredenburg 2008), with > 32% of species threatened and at least 43% experiencing  population declines (Stuart et al. 2004). Chemical pollution is a concern for the health of both amphibians and humans. It is considered the second greatest threat (behind habitat loss) to aquatic and amphibious species in the United States and has been linked to amphibian declines and disease (Davidson et al. 2002; Rohr et al. 2008a). Similarly, contaminants have been linked to mortality and disease in humans (Dietert et al. 2010). Importantly, many vital systems of amphibians, such as endocrine and immune systems, are similar to those in humans (Hayes 2005), and a genome analysis revealed that the amphibian *Xenopus tropicalis* has > 1,700 genes with human disease associations (Hellsten et al. 2010). Thus, in addition to being of conservation concern, amphibians might be an underused model taxon for studying stressor effects on human health.

Although the hypothesis that contaminants are a factor in amphibian declines is plausible, most previously tested chemicals have not directly killed amphibians at or below expected environmental concentrations (EECs; but see Rohr et al. 2006b; Storrs and Kiesecker 2004), although sublethal and indirect effects can be strong (Rohr et al. 2006a). Nevertheless, many chemicals remain untested on amphibians. For example, chlorothalonil is the most commonly used synthetic fungicide in the United States (Kiely et al. 2004) and is toxic to shrimp, insects, and fish at or below the EEC (164 μg/L) (Caux et al. 1996; Grabusky et al. 2004). It can be transported great distances and has been found in tropical mountains where most amphibian declines have occurred (Stuart et al. 2004). However, its effects on amphibians have rarely been studied.

Chlorothalonil can also affect vertebrate and invertebrate immune systems. Chlorothalonil exposure was associated with contact dermatitis (Penagos 2002) and DNA damage to leukocytes of farmers 1 day after spraying (Lebailly et al. 1997). It can be immunosuppressive to oysters and fish, reducing macrophage viability and function, immunoglobulin M, and expression of proinflammatory cytokines (Baier-Anderson and Anderson 2000; Shelley et al. 2009). These findings are a concern because pollution is often associated with wildlife disease emergence (Dobson and Foufopoulos 2001) and amphibians are being decimated by infectious disease (Daszak et al. 2003). The objective of this study was to quantify the effects of chlorothalonil on amphibian survival, immunity, corticosterone levels, and liver density.

Chlorothalonil (2,4,5,6-tetrachloroisophthalonitrile) is widely used to control fungus on peanuts, corn, and potatoes (Cox 1997). Approximately 14 million pounds are applied annually in the United States, with approximately 500,000 pounds used per year in Florida [U.S. Environmental Protection Agency (EPA) 1999], the location of the present study. Chlorothalonil is typically applied during the wet season, corresponding to the breeding activity of many amphibians (Rohr et al. 2004).

Chlorothalonil binds to glutathione, which disrupts cellular respiration (Grabusky et al. 2004), a vital process for virtually every organism, including humans. In water, chlorothalonil is short lived, with a half-life of approximately 44 hr (U.S. EPA 1999). The primary chlorothalonil metabolite (4-hydroxy-2,5,6-trichloroisophthalonitrile) is estimated to be 30 times more acutely toxic than chlorothalonil and is also more persistent and mobile in soil (U.S. EPA 1988). During its manufacture, chlorothalonil is also contaminated with hexachlorobenzene (Hung et al. 2010), a probable carcinogen with a soil half-life of 3–6 years (Cox 1997).

Shuman et al. (2000) detected chlorothalonil concentrations of ≤ 290 μg/L in runoff, and chlorothalonil has been detected in groundwater (“standpipe” wells) at concentrations ≤ 272 μg/L. Nevertheless, the EEC of chlorothalonil in ponds [calculated using the U.S. EPA’s GENEEC software, version 2; for parameters, see Supplemental Material, Table S1(doi:10.1289/ehp.1002956)] is approximately 164 μg/L. If lowest observable effect concentrations (LOECs) of a chemical are near or below its EEC, then it poses sufficient risk to warrant higher-level modeling. Hence, effects of a chemical near or below the EEC can affect the decision to approve its use.

## Materials and Methods

This work was approved by animal care and use committees of the University of South Florida (W3228) and the University of Florida (023-08WEC). All animals used were treated humanely and with regard for alleviation of suffering.

*Mesocosm experiment.* The mesocosm experiment was conducted at the University of Florida’s Gulf Coast Research and Education Center during July and August 2008 (35 days total). Mesocosms consisted of cattle water tanks (1.8 m diameter, 60 cm deep, ~ 1,100 L) containing 800 L water, 300 g leaf litter, and local zooplankton, phytoplankton, periphyton, insect, gastropod, and crayfish species [see Supplemental Material, Table S2 (doi:10.1289/ehp.1002956)]. Mesocosms were covered with 60% shade cloth to prevent overheating and entry or escape of animals. Each tank received 10 *Rana sphenocephala* (southern leopard frog) tadpoles from eight clutches (collected at N 28°06.759´, W 082°23.014´) and 25 *Osteopilus septentrionalis* (Cuban treefrog) tadpoles (all at Gosner stages 25–28; Gosner 1960) from five clutches (collected at N 28°03.537´, W 082°25.410´).

Tanks were arranged in a randomized block design with four replicates of each treatment (a total of 16 tanks). There were two control treatments, receiving either 50 mL of water or 50 mL acetone solvent (used to dissolve chlorothalonil). Tanks for the remaining two treatments received chlorothalonil (technical grade, purity > 98%; Chemservice, West Chester, PA) dissolved in 50 mL acetone so that nominal concentrations in the tanks were either one time the EEC (1×; 164 μg/L) or two times the EEC (2×; 328 μg/L). Tanks were dosed the same day as the amphibians were added, and targeted nominal concentrations closely matched the actual concentrations (1×, 172 μg/L; 2×, 351 μg/L; spiked recovery efficiencies, 95%). Thus, for simplicity and consistency across the experiments in this article, we refer to the nominal concentrations. Several water quality and chemistry variables were quantified at various times during the experiment [see Supplemental Material, “Mesocosm Experimental Methods” and Tables S3 andS4 (doi:10.1289/ehp.1002956)]. Standardized dip net sampling of each tank was conducted the third day of the experiment to quantify any rapid mortality associated with chlorothalonil exposure. The number of metamorphosed frogs was noted daily, and tadpole survival was determined 5 weeks after dosing.

*Laboratory experiment I.* We obtained *Hyla squirella* and *O. septentrionalis* from multiple, thoroughly mixed clutches collected from two adjacent retention ponds in Tampa, Florida, in July 2008 (N 28°0.322´, W 82°19.532´). We employed a completely randomized design with 21 32-L glass aquaria, each filled with 10 L artificial spring water (Cohen et al. 1980), with water hardness of 62.7 ppm (5B Hardness Test Kit; HACH Co., Loveland, CO) and pH ~ 7.0). Aquaria were maintained in a laboratory at the University of South Florida at 27°C and on a 14:10-hr light:dark cycle. Each aquarium received five *H. squirella* and 15 *O. septentrionalis* tadpoles (Gosner stages 25–28), and tadpoles were fed boiled organic spinach daily. We used five treatments of technical grade chlorothalonil (purity > 98%; Chemservice; actual concentrations, 0.176, 1.76, 17.6, 176, and 1,760 μg/L) and two control treatments [water and solvent (500 ng/L acetone)], with three replicates per treatment. The targeted nominal concentration for the chlorothalonil stock was 1,640 μg/L, and the actual concentration was 1,760 μg/L (spiked recovery efficiencies, 95%). All of the other concentrations were attained through serial dilutions of this stock solution. Again, for simplicity and consistency across the experiments, we refer to the nominal concentrations. We quantified frog survival and preserved dead tadpoles 12 hr after the start of the experiment and then every 24 hr for 4 days (there were no water changes). Surviving tadpoles were euthanized and preserved (70% ethanol) at the end of the experiment.

*Laboratory experiment II.* The same protocols used in laboratory experiment I were used in this experiment, conducted in October 2008, with the following exceptions. We tested three tadpole species: *R. sphenocephala*, *O. septentrionalis*, and *H. cinerea* (all starting at Gosner stage 25). We employed a completely randomized design with 144 500-mL mason jars, each filled with 300 mL artificial spring water and each receiving three tadpoles of a single species. Species were isolated in this experiment because *O. septentrionalis* was occasionally observed depredating *H. squirella* in laboratory experiment I. The jars received one of six chlorothalonil treatments (0.0164, 0.164, 1.64, 16.4, 82.0, or 164 μg/L) or water or solvent. We used the same stock solution as in laboratory experiment I. A single water change occurred on day 7, and each jar was redosed at that time. There were six replicates per species per treatment. The number of surviving tadpoles was noted after 4 hr, 24 hr, and then every 24 hr, for 10 days, and all dead tadpoles were removed and preserved in formalin at those times.

To quantify the effects of chlorothalonil on tadpole livers and immune cells, at the end of the experiment one arbitrarily selected *O. septentrionalis* from each replicate was euthanized, embedded in paraffin, sectioned, and stained with hematoxylin and eosin. We used *O. septentrionalis* for liver, immune, and corticosterone quantification because it had the lowest mortality of the three species and thus offered us the most survivors per tissue. To test whether chlorothalonil exposure affected liver tissue integrity, we used ImageJ64 software (Rasband 2010) to calculate liver tissue density, following ImageJ’s *Quantifying Stained Liver Tissue* (Burger and Burge 2009), which reports the density of stained tissue within a designated area. To test whether chlorothalonil exposure affected density of liver immune cells, we counted the number of melanomacrophages and granulocytes per field of view at 400× magnification. Melanomacrophages and granulocytes are leukocytes that help defend against a variety of parasites (Rohr et al. 2008b). Because of the morphological similarity among granulocytes, we conservatively categorized all granule- containing immune cells as granulocytes, but most were likely eosinophils.

*Corticosterone experiment.* We used *O. septentrionalis* tadpoles (Gosner stages 25–28; the same population as used in laboratory experiment II) to quantify the effect of chlorothalonil exposure on frog corticosterone levels, a steroid hormone elevated in response to natural and anthropogenic stressors, including pesticides (Martin et al. 2010). We used the same general protocols as described in laboratory experiment II and the following treatments: 0.164, 16.4, 82, and 164 μg/L chlorothalonil, and water and solvent controls. These treatments were crossed with one of three chlorothalonil exposure durations: 4, 28, or 100 hr (*n* = 3, 2, and 3, respectively). The exception, however, was that tadpoles exposed to 164 μg/L chlorothalonil were only exposed for 4 hr because they did not survive for 28 or 100 hr of exposure. This design resulted in 43 independent replicates. After the appropriate exposure duration, tadpoles were euthanized, and individual tadpoles were weighed (to 0.0001 g) and homogenized in ultrapure water. Tritiated corticosterone (2,000 cpm) was then added to each sample to quantify recoveries postextraction. We used a corticosterone enzyme immunoassay (EIA) kit (catalog no. 900-097; Assay Designs, Ann Arbor, MI) to quantify hormone levels in each sample. Individual recoveries (mean, 55.3%) and tadpole mass measurements were used to estimate corticosterone per gram of tadpole tissue. Detailed methods for this EIA kit and a discussion of its potential limitations are provided in Supplemental Material (doi:10.1289/ehp.1002956).

*Statistical analyses.* For all experiments and responses, we compared the water and solvent controls. Because we found no difference between these treatments (*p* > 0.328), we pooled the two treatments into one “control” group for all subsequent analyses.

For the mesocosm experiment, all analyses were conducted on the arcsine-square-root–transformed proportion of *R. sphenocephala* and *O. septentrionalis* surviving to the end of the experiment, controlling for the four spatial blocks. We tested whether chlorothalonil was associated with mortality relative to the control treatments by conducting a permutation-based multivariate regression analysis. For the laboratory experiment, we arcsine-square-root transformed the proportion of tadpoles surviving until the end of the experiment and log transformed hours to death, mass of survivors, amount of liver damage, and melanomacrophage and granulocyte counts to meet parametric assumptions. For the liver and immune analyses, we log transformed chlorothalonil concentration and weighted the time to death analyses by the number of animals that died per replicate. If a dose response appeared linear, chlorothalonil concentration was treated as a continuous predictor in a regression model (liver density). If a dose response was nonlinear but relatively simple (one inflection point), chlorothalonil concentration was treated as a continuous predictor, and we used polynomial regression with type II sums of squares to fit the data (immune responses). If a response was nonlinear and relatively complex (more than one apparent inflection point), chlorothalonil concentrations were treated as levels of a categorical predictor followed by Fisher’s least significant difference (LSD) multiple comparison test to determine which levels were different from one another (proportion of tadpoles that survived and time to death). As an additional test for nonmonotonicity (hump-shaped dose response), we eliminated the highest concentrations, which typically caused considerable mortality, and used polynomial regression to test for a quadratic dose–response relationship with the remaining concentrations. For the immune responses, we conducted a multivariate polynomial regression model with melanomacrophages and granulocytes as responses and followed it by univariate analyses on each response variable. We log-log transformed these relationships to improve fit and meet the assumption of the polynomial regression.

For the corticosterone experiment, we conducted polynomial regression (using least trimmed squares) with chlorothalonil concentration as a continuous predictor and log- transformed corticosterone as the response variable. All statistical analyses were conducted with Statistica (version 8.0; Statsoft, Tulsa, OK). We did not calculate LC_50_ (concentration that results in death of 50% of individuals by a given time) values for any responses because all three dose–response experiments showed evidence of nonmonotonicity, which would violate the assumptions of LC_50_ calculations.

## Results

**Figure 1:**
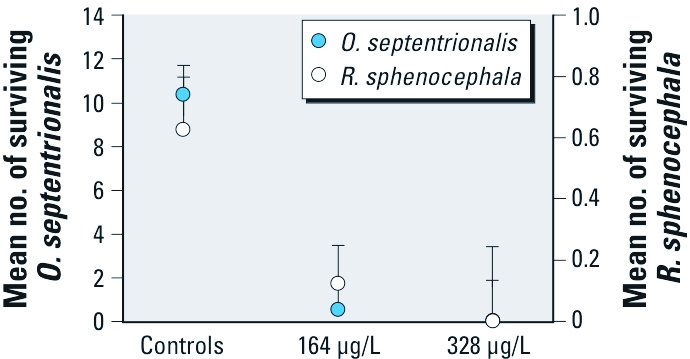
Survival of tadpoles in the mesocosm experiment shown by the number of *O. septentrionalis* and *R. sphenocephala* tadpoles surviving after exposure to measured concentrations of chlorothalonil (1× EEC, ~ 164 μg/L; 2× EEC, ~ 328 μg/L; single pulse) relative to controls (water and solvent combined). Both species had 0% survival at 328 μg/L. For *O. septentrionalis* , *n* = 25/treatment; for *R. sphenocephala*, *n* = 10/treatment.

*Mesocosm experiment.* The multivariate permutation test revealed a positive association between chlorothalonil concentration and amphibian mortality (*p* = 0.005), with controls having less mortality than both the 164 μg/L (*p* = 0.013) and 338 μg/L chlorothalonil treatments (*p* = 0.023; Figure 1). Chlorothalonil concentration was positively associated with the mortality of both *O. septentrionalis* (*p* = 0.001) and *R. sphenocephala* (*p* = 0.064; Figure 1).

A mean (± SE) of 1.5 ± 0.327 live tadpoles  were captured per dip netting session in control tanks, but no live tadpoles were netted from chlorothalonil tanks (the only tanks where dead tadpoles were netted). These results suggest that most of the mortality associated with chlorothalonil occurred within the first 72 hr of exposure.

**Figure 2:**
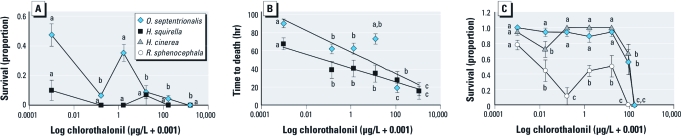
Survival of tadpoles in laboratory experiments I and II. Survival (*A*) and time to death (*B*) of *O. septentrionalis* (15 tadpoles/tank) and *H. squirella* (5 tadpoles/tank) exposed to several concentrations of chlorothalonil (0.164, 1.64, 16.4, 164, and 1,640 μg/L) and controls (water and solvent combined) for laboratory experiment I (*n* = 3 for all chlorothalonil concentrations; *n* = 6 for controls). (*C*) Survival of *O. septentrionalis*, *H. cinerea*, and *R. sphenocephala* exposed to several concentrations of chlorothalonil (0.0164, 0.164, 1.64, 16.4, 82.0, and 164 μg/L) and control treatments (water and solvent combined) for laboratory experiment II (*n* = 6 for all chlorothalonil concentrations: *n* = 12 for controls). Values shown are mean ± SE. Different lowercase letters indicate that responses for a given species were significantly different (*p* < 0.05) among treatment levels according to Fisher’s LSD multiple comparison tests.

*Laboratory experiment I.* Survival was lower for *H. squirella* than for *O. septentrionalis*, most likely because *O. septentrionalis* depredated *H. squirella* (Figure 2A). Despite this predation, time to death for *H. squirella* was shorter for tadpoles exposed to any chlorothalonil concentration relative to controls [Fisher’s LSD, *p* < 0.023 for controls compared with any chlorothalonil concentration (Figure 2B); for full analysis of covariance results, see Supplemental Material (doi:10.1289/ehp.1002956)].

For *O. septentrionalis*, survival was nonmonotonic, with low and high concentrations causing significantly greater mortality than intermediate concentrations and control treatment (Figure 2A). Relative to controls, survival was reduced by > 80% in the 0.164, 17.6, 164, and 1,640 μg/L concentrations, but survival was not significantly reduced by 1.64 μg/L chlorothalonil, and this concentration was significantly different from both adjacent concentrations (Figure 2A). This nonmonotonicity was also supported by polynomial regression, which produced a significant quadratic term for concentrations < 16.4 μg/L [for statistics, see Supplemental Material (doi:10.1289/ehp.1002956)]. Relative to controls, time to death was shorter for *O. septentrionalis* tadpoles exposed to any chlorothalonil concentration (Fisher’s LSD, *p* < 0.021 for 0 μg/L vs. 0.164, 1.64, 164, or 1,640 μg/L; Figure 2B), with the exception of 16.4 μg/L (Fisher’s LSD, *p* = 0.190; Figure 2B).

*Laboratory experiment II.* For each species, the 164 μg/L concentration killed 100% of the tadpoles by the end of the experiment [Figure 2C; for mortality through time and full statistical results, see Supplemental Material, Figure S1 and methods for laboratory experiment II, respectively (doi:10.1289/ehp.1002956)]. However, we observed considerable variation among species in their sensitivity to chlorothalonil. *R. sphenocephala* appeared most sensitive, experiencing 86% mortality at 0.164 μg/L and 100% mortality at 82 μg/L (Figure 2C), whereas *O. septentrionalis* was least sensitive (Figure 2A).

The dose response for survival was significantly nonmonotonic for *R. sphenocephala* and *H. cinerea*, with low and high concentrations causing significantly greater mortality than intermediate concentrations and control treatment (Figure 2C), a result similar to the nonmonotonic dose response revealed in laboratory experiment I for *O. septentrionalis*. For *R. sphenocephala*, 0.164 μg/L caused significantly more mortality than did each adjacent concentration, and we found a significant quadratic term for the response to doses < 82 μg/L. For *H. cinerea*, 0.0164 μg/L caused significantly more mortality than did each adjacent concentration, and as for *R. sphenocephala*, we found a significant quadratic term for the response to doses < 16.4 μg/L [for polynomial results for both species; see Supplemental Material (doi:10.1289/ehp.1002956)]. As a reminder, each data point in Figure 2C is the mean of six data points, and thus the 0.0164 μg/L concentration for *H. cinerea* is not an outlier or artifact.

*O. septentrionalis* did not exhibit a nonmonotonic response in this experiment as it did in laboratory experiment I (Figure 2A,C). This is possibly due to differences in tadpole densities, developmental stages, source populations, or bioaccumulation of chlorothalonil associated with *O. septentrionalis* depredating *H. squirella* in laboratory experiment I. Chlorothalonil has been documented to bioaccumulate up to 3,000 times in fish (Cox 1997; U.S. EPA 1999).

**Figure 3:**
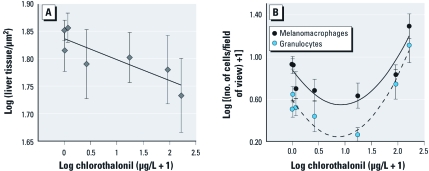
Effect of chlorothalonil on tadpole liver health and immunity. Density of liver tissue (*A*) and number of melanomacrophages and granulocytes in the liver (*B*) of *O. septentrionalis* tadpoles exposed to several concentrations of chlorothalonil [0.0164 (*n* = 9), 0.164 (*n* = 4), 1.64 (*n* = 6), 16.4 (*n* = 5), 82.0 (*n* = 5), and 164 μg/L (*n* = 3) and controls (water and solvent combined (*n* = 6)]. Values shown are mean ± SE and best-fit lines.

Increasing chlorothalonil concentrations were associated with significant decreases in liver density of *O. septentrionalis* [*F*_1,40_ = 4.82, *p* = 0.034; Figure 3A; see also Supplemental Material, Figure S2 (doi:10.1289/ehp.​1002956)]. Chlorothalonil concentration was also associated quadratically with both melanomacrophages and granulocytes in this species (Figure 3B; for statistics, see Supplemental Material). That is, relative to controls, tadpoles exposed to low concentrations had fewer of these immune cells, whereas tadpoles exposed to high concentrations had elevated numbers of these cells (Figure 3B). We observed considerable mortality at the 82 and 164 μg/L concentrations that may have confounded our immune results and might explain the increase in immune cells at these concentrations. Thus, we reanalyzed the dose response excluding these two highest concentrations and discovered that, at these lower and more environmentally common concentrations, chlorothalonil was associated with a reduction in both melanomacrophages (*F*_1,32_ = 4.67; *p* = 0.038) and granulocytes (*F*_1,32_ = 5.52; *p* = 0.025; Figure 3B).

**Figure 4:**
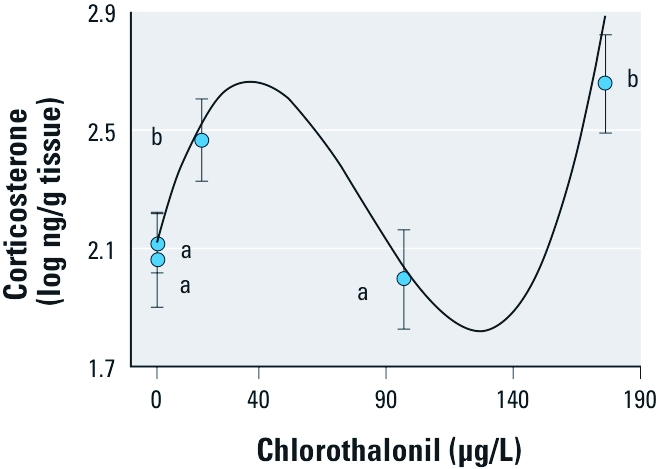
Effects of chlorothalonil on corticosterone per gram of *O. septentrionalis* tissue shown as least squares means ± 1 SE. Means were averaged across the three chlorothalonil exposure durations (4, 28, and 100 hr), except for the 164 μg/L concentration, where only the 4 hr duration mean is shown because longer exposure killed the tadpoles. Also shown is the significant third-order polynomial function (*y* = 1.886571 + 0.035582*x* – 0.000668*x*^2^ + 0.000003*x*^3^) for the relationship between chlorothalonil concentration and log corticosterone, adjusted for the effect of exposure duration. The corticosterone level for the 164 μg/L concentration is underestimated because it is the only mean based on 4 hr, rather than an average of 44 hr, of chlorothalonil exposure, and corticosterone increased significantly and log-linearly with the duration of chlorothalonil exposure (coefficient for log exposure duration = 0.269). Concentrations with different lowercase letters are significantly different from one another by Fisher’s LSD multiple comparison test (*n* = 13, 5, 7, 6, and 2 for 0, 0.164, 16.4, 82.0, and 164 μg/L, respectively).

*Corticosterone experiment.* Corticosterone levels increased significantly with chlorothalonil exposure duration (*F*_1,27_ = 11.57, *p* = 0.002). After 4 hr exposure to chlorothalonil, the relationship between log corticosterone levels and chlorothalonil concentration was significantly nonlinear (concentration^3^: *F*_1,11_ = 6.12; *p* = 0.031), with low and high concentrations of chlorothalonil being associated with higher levels of corticosterone than were intermediate concentrations and controls (Figure 4). Multiple comparison tests further supported the conclusion that this dose–response curve was significantly nonlinear (Figure 4). This same general pattern persisted for up to 100 hr of exposure, but tadpoles were not available after the 4 hr exposure duration for 164 μg/L because of high mortality (Figure 4). As a reminder, we conducted this study on the *O. septentrionalis* population that did not exhibit any significant nonmonotonic mortality response to chlorothalonil and exhibited significant mortality only at concentrations ≥ 82 μg/L (Figure 2C).

## Discussion

Ultimately, scientists should use a weight-of-evidence approach to evaluate risk posed by chemicals, which is partly why we conducted four experiments to quantify the effects of chlorothalonil on amphibians: a contrived, but highly controlled, laboratory experiment (laboratory experiment II), a more ecologically relevant laboratory experiment where we allowed species to interact (laboratory experiment I), a laboratory experiment to assess whether corticosterone levels exhibited a dose response similar to that for mortality (corticosterone experiment), and a field mesocosm experiment with a complex freshwater community (mesocosm experiment). In all of these experiments, we found adverse effects of chlorothalonil on tadpoles. Although in laboratory experiment I we had low survival of *H. squirella* in the control group, possibly because of depredation by *O. septentrionalis*, these species regularly coexist, making this interaction ecologically relevant. This experiment also reinforced the significant lethality of the EEC and lower concentrations of chlorothalonil and provided the first indication of a nonmonotonic dose–mortality response for this pesticide (Figure 2A). We conducted a follow-up experiment using three amphibian species, this time preventing heterospecific interactions. This experiment had 80–100% survival of the control tadpoles, simplifying data interpretation. It revealed that all three species were highly susceptible to chlorothalonil, with the EEC causing 100% mortality of each species in < 10 hr of exposure. Moreover, in this experiment, we found evidence of nonmonotonic dose responses for mortality and full-body measurements of corticosterone, with low and high levels elevating both responses. Finally, in our mesocosm study, both the 164 and 328 μg/L concentrations significantly reduced amphibian survival, suggesting that the laboratory results might be relevant to effects in nature. Together, these four experiments indicate that amphibians, in general, are susceptible to the EEC of chlorothalonil and that even low concentrations can cause amphibian mortality and physiological stress responses.

Our finding that amphibians are sensitive to chlorothalonil is consistent with studies examining the sensitivity of aquatic vertebrates and invertebrates to chlorothalonil. For several fish species, 48- and 96-hr LC_50_ values are < 20 μg/L and LOECs are near 1 μg/L chlorothalonil (Caux et al. 1996). The 48-hr LC_50_ for *Bufo bufo japonicas* was 160 μg/L (Hashimoto and Nishiuchi 1981). *Daphnia magna* had delayed reproduction when exposed to 32 μg/L (Ernst et al. 1991); in fathead minnows ≥ 6.5 μg/L chlorothalonil decreased the number of eggs per spawn, egg hatchability, and fry survival (as cited by Grabusky et al. 2004). The LOEC for *H. cinerea* and *R. sphenocephala* survival in our study was 10,000 times less than the EEC (0.0164 μg/L; Figure 2C) and was the lowest concentration we tested. Hence, we did not test low enough concentrations to detect a no observable effect concentration for the survival of these two species.

Three of the four amphibian species that we tested showed evidence of a nonmonotonic dose–mortality response to chlorothalonil (*O. septentrionalis*, Figure 2A; *H. cinerea* and *R. sphenocephala*, Figure 2C), with low and high levels causing significantly greater mortality than did intermediate levels and controls. Furthermore, for all species and experiments, the low-dose increase in mortality occurred within a single order of magnitude (either 0.016 or 0.16 μg/L). Although the nonmonotonic dose response for survival was observed for *O. septentrionalis* in only one of the two experiments (laboratory experiment I; these experiments used different conditions and source populations), in the experiment where *O. septentrionalis* did not exhibit a nonmonotonic dose response for survival (laboratory experiment II), it did exhibit a nonmonotonic dose response for corticosterone. Hence, the nonmonotonic response was consistent and reproducible both within and across species, but whether low-dose exposure to chlorothalonil and the associated stress response cause mortality appears to be context dependent. Nonmonotonic responses are important because they defy the traditional toxicological assumption that higher concentrations of a contaminant always cause greater harm. Nonmonotonic patterns have been observed previously in response to chlorothalonil (Shelley et al. 2009) and other agrochemicals (Storrs and Kiesecker 2004). Nonmonotonic responses can be caused by multiple mechanisms, affecting responses differently at different doses, or by endocrine disruption (Welshons et al. 2003). Indeed, the Canadian Wildlife Service concluded that chlorothalonil might qualify as an endocrine disruptor because it has the potential to interfere with endogenous hormones and enzymes and is an immunomodulator (Grabusky et al. 2004). However, the mechanism or mechanisms underlying nonmonotonic dose responses in this study remain unknown.

In addition to mortality, chlorothalonil was associated with immunomodulation of the surviving *O. septentrionalis* tadpoles. This finding is consistent with DNA damage to mononuclear leukocytes of farmers 1 day after spraying chlorothalonil (Lebailly et al. 1997) and with studies of chlorothalonil-induced immunosuppression of fish and marine invertebrates (Baier-Anderson and Anderson 2000). Increases in chlorothalonil concentration up to 17.6 μg/L, concentrations to which humans are commonly exposed (Daly et al. 2007), were associated with reduced liver granulocytes and melanomacrophages in tadpoles, whereas further increases in chlorothalonil caused increased numbers of liver granulocytes and melanomacrophages (Figure 3B). This increase in immune cells might be in response to chlorothalonil-induced liver damage, based on our observations of decreased *O. septentrionalis* liver density at these higher concentrations [see Supplemental Material, Figure S2 (doi:10.1289/ehp.1002956)]. Alternatively, the increase in immune cells might itself have contributed to liver damage, because high levels of melanomacrophages and granulocytes can cause tissue damage (Rose et al. 1999). Although not yet studied, it is possible that exposure to chlorothalonil could reduce tolerance and resistance to parasites, which has been shown for wildlife and humans exposed to other agrochemicals (Dietert et al. 2010; Rohr et al. 2008b). If so, this could further reduce tadpole survivorship.

To our knowledge, we provide the first evidence that chlorothalonil elevates corticosterone. The significant nonmonotonic dose response of corticosterone to chlorothalonil was qualitatively similar to the mortality responses we observed in this study, underlining the consistent presence of nonmonotonic responses to this chemical. However, we do not know the direction of causation. Approaching mortality could have resulted in a systemic stress response that altered corticosterone and immune parameters; changes in corticosterone and immune parameters could have caused the mortality; or both of these scenarios could have occurred. Mortality at the highest concentrations of chlorothalonil seemed to occur too quickly to be mediated by corticosterone. However, it is plausible that corticosterone could have been involved in the mortality and immune cell changes observed at low concentrations of chlorothalonil. First, corticosterone is known to cause elevations in circulating granulocytes in other animals (Davis et al. 2008), either by inducing proliferation or by efflux from cell reservoirs. Second, continuously elevated corticosterone has manifold negative effects on health, including muscle atrophy, reduced neurogenesis, and immune suppression or dysregulation (Martin 2009). Lastly, glucocorticoids, including corticosterone, are commonly elevated in response to stressors, natural and anthropogenic (Martin et al. 2010), and even in cases where elevations are insufficient to cause mortality, they can generally compromise health, even in humans (Wingfield and Sapolsky 2003). Although we cannot say with certainty whether the immunological effects observed in this study were mediated by corticosterone, we strongly advocate future efforts to assess the role of chlorothalonil and glucocorticoids as potential endocrine disruptors, especially as disruptors of the immune system and disease resistance.

Although pesticides have been suggested as a cause of amphibian declines, there are few convincing cases in which pesticides cause high enough mortality at environmentally realistic concentrations to directly affect amphibian populations (Belden et al. 2010; Rohr et al. 2006b). Sometimes even high mortality of larval amphibians can have little observable effect on the population because of density-mediated compensation, in which survivors of a factor experience lower mortality than do control animals after the stressor is removed because of less competition for resources (Rohr et al. 2006b). However, based on amphibian demographic models  that incorporate negative density dependence (Vonesh and De la Cruz 2002), the level of EEC-induced mortality reported here would likely reduce amphibian population sizes. Given that chlorothalonil caused nearly 100% mortality at the EEC, caused significant mortality at concentrations four orders of magnitude below the EEC, and caused immunomodulation in surviving individuals, exposure to this chemical has the potential to both directly and indirectly cause amphibian declines. Indeed, frog die-offs have been reported after chlorothalonil applications to cranberry bogs (Winkler et al. 1996), and in neotropical montane regions where amphibians are declining, chlorothalonil has been regularly detected at levels that caused significant mortality in the present study (Daly et al. 2007). This makes chlorothalonil a plausible contributor to declines, although additional work is needed to demonstrate a causal link. Given these findings and similarities between the vital systems of amphibians and humans, we encourage future studies to quantify the effects of chlorothalonil on amphibian populations and human health.

## Supplemental Material

(604 KB) PDF
