# Supplementary material for: The Fungicide Chlorothalonil Is Nonlinearly Associated with Corticosterone Levels, Immunity, and Mortality in Amphibians
Source: Environ Health Perspect. 2011 Apr 4;119(8):1098–103. doi: 10.1289/ehp.1002956 (PMC3237349; doi:10.1289/ehp.1002956)
Supplement: (604 KB) PDF [file ehp.1002956.s001.pdf]

Supplemental Materials for:

Title: The Fungicide Chlorothalonil is Nonlinearly Associated with Corticosterone Levels, Immunity, and Mortality in Amphibians

Authors: Taegan McMahon, Neal Halstead, Steve Johnson, Thomas R. Raffel, John M. Romansic, Patrick W. Crumrine, Raoul K. Boughton, Lynn B. Martin, and Jason R. Rohr

Table of Contents:

	Page
Table S1	2
Mesocosm Experiment Methods	2-3
Tables S2, S3 and S4	3-4
Lab Experiment I Methods and Statistical Analysis	4
Lab Experiment II Methods and Statistical Analysis	4-5
Corticosterone Experiment Methods	5-6
Figure S1 and Figure S2	7-8

Table S1. Parameter values used in the USEPA GENEEC v2 software to calculate a generalized expected environmental concentration (EEC).

GENEEC Parameter	Chlorothalonil
depth of incorporation (0-6 in)	0
spray quality	fine: EFED[a]
nozzle height (in.)	20-50: EFED[a]
Trade name	Bravo Weather Stik
Crop	potato
Rate (pounds of active ingredients/acre taken from specimen label)	1.25
Number of applications	9
Times between applications	5
koc	1380[b]
Soil half-life (days)	90[b]
Wetted application?	Yes
Application method	Ground spray[a]
No spray zone (feet)	0
Solutbility (mg/L)	0.6[b]
Aquatic half-life (days)	8.8[d]
Hydrolysis half-life (days)	38[d]
Photolysis half-life (days)	-
Peak EEC (μg/L)	164

[a] Default in the program

[b] http://extoxnet.orst.edu/

[c] USDA

[d] Spectrum Laboratories Inc., http://www.speclab.com/

Mesocosm Experiment Methods

Zooplankton and algal inoculations occurred three weeks before the start of the experiment. Tadpole, insect, gastropod, and crayfish were housed in artificial spring water in aquaria in the laboratory, until addition to the mesocosms at the start of the experiment (the day pesticides were applied). All of the organisms added to the tanks were collected from ponds within 1 km of N28°06.759' W082°23.014', near the transition of suburban and rural environments but not near any agriculture. Tadpole densities used in this experiment were selected to match those commonly observed in the field (pers. obs. by Taegan McMahon).

Standardized dip net sampling of each tank was conducted three days after the start of the experiment to quantify any rapid mortality associated with the treatments. Three samples were collected from, and returned to, each tank and separate nets were used for each treatment. To avoid transfer of microrganisms among tanks within each treatment, nets were also thoroughly disinfected after sampling each tank (potassium permanganate treatment followed by rinses with Novaqua® and tap water).

The Hillsborough County Water Resource Services Environmental Laboratory (15610 Premiere Dr. Tampa, FL 33624) also quantified nitrogenous compounds, total nitrogen, total phosphorous, and calcium collected from control tanks at the start of the experiment (data in Table S3). Dissolved oxygen, pH, and temperature were quantified at dawn and dusk from every

tank during experimental weeks two and four (Corning 313 pH/temp Meter and Ecosense YSI DO 2000 (EcoEnvironmental, Perth, Australia) data provided in Table S4).

We saw high control mortality in the *Mesocosm Experiment*; this may have been due to predation in the tanks. This mortality was accounted for in all of the analyses run, and high mortality is often seen in natural settings.

Table S2. Organisms added to the each cattle tank in the beginning of the mesocosm experiment excluding zooplankton and algae.

Taxa	Amount
Amphibians	
Rana sphenocephala tadpoles	10
Osteopilus septentrionalis tadpoles	25
Arthropods	
Anax junius nymphs	2
Belostoma flumineum adults	2
Water scorpion adults	3
Libellulid nymphs	1
Crayfish juveniles	3
Corixidae sp. adults	10
Gastropods	
Planorbella trivolvis adults	9
Planorbella scalaris adults	10
Viviparus georgianus adults	10
Melanoides tuberculata adults	20
Pomacea caniculata adults	6
Plants	
Hydrilla verticillata	250 g
Utricularia macrorhiza	250 g

Table S3. Quantified water quality measurements from pooled water samples collected from control tanks at the start of the Mesocosm experiment.

Water Quality Measurement (μg/L)	Control
Calcium	39000
Nitrate	77
Nitrite	61
Total Kjeldahl Nitrogen	30
Total Nitrogen	370
Total Phosphorous	60

Table S4. Mean values (±SE) for dissolved oxygen, pH, and temperature; quantified at dawn and dusk during experimental weeks two and four in the Mesocosm experiment.

	Control (n=8)				172 µg/L Chlorothalonil (n=4)				351 µg/L Chlorothalonil (n=4)			
	Dawn Mean	±SE	Dusk Mean	±SE	Dawn Mean	±SE	Dusk Mean	±SE	Dawn Mean	±SE	Dusk mean	±SE
DO (ppm)	3.98	0.38	4.66	0.61	4.39	0.28	5.87	0.33	4.21	0.41	4.88	0.52
pH	7.60	0.07	7.69	0.23	7.68	0.09	7.75	0.14	7.67	0.11	7.65	0.18
Temperature (^{o}C)	28.16	0.20	29.93	1.21	28.13	0.14	30.31	0.14	28.04	0.17	30.03	0.23

Laboratory Experiment I: Methods and Statistical Analysis

The chemical concentration of the chlorothalonil stock (stock used for serial dilutions) was verified by Mississippi State Chemical Lab (Box CR Miss. State, MS 39762; spiked recovery efficiencies: 95%). All main effects and interactions were significant for percent survival (Species: $F_{1,30}$=25.96, P<0.001; Chlorothalonil: $F_{5,30}$=8.06, P<0.001; Species x chlorothalonil: $F_{5,30}$=3.82, P=0.009; Figure 2A) and time to death (Species: $F_{1,30}$=9.52, P=0.004; Chlorothalonil: $F_{5,30}$=35.21, P<0.001; Species x chlorothalonil: $F_{5,30}$=3.30, P=0.017; Figure 2B). For *O. septentrionalis*, survival was non-monotonic with low and high concentrations causing significantly greater mortality than intermediate concentrations and controls (Fig. 2A). This non-monotonicity was supported by polynomial regression, which produced a significant quadratic term for concentrations <16.4µg/L (log chlorothalonil: $F_{1,9}$=22.04, P=0.001, log chlorohtalonil^2: $F_{1,9}$=22.02, P=0.001).

Laboratory Experiment II: Methods and Statistical Analysis

Rana sphenocephala and *H. cinerea* were collected from multiple clutches from the same pond as described in *Laboratory Experiment I*, but *O. septentrionalis* tadpoles were obtained from three clutches oviposited in water-filled wading pools left uncovered in a fenced section of the University of South Florida Botanical Gardens (N28^{o}03.537' W082^{o}25.410'). Tadpoles from multiple clutches of each species were thoroughly mixed before being distributed among replicates in the experiment. Dissolved oxygen was not quantified because, in our experience, the small volume (300 mL) and high surface area to volume ratio provides close to saturated dissolved oxygen conditions.

No individuals survived in the 176µg/L treatment, and their bodies were too degraded to be sectioned and stained for liver quantification. Hence, we exposed six individually-housed *O. septentrionalis* tadpoles to 164 µg/L of chlorothalonil for just seven hours (using the same procedures as in *Laboratory Experiment II*), a sublethal exposure duration. These individuals were euthanatized and their bodies were embedded, sectioned, and H&E stained at Moffitt Cancer Center &Research Institute (12902 Magnolia Dr. Tampa, FL 33612; as described in main text).

For *Laboratory Experiment II*, we fit third-order polynomial functions to the full dose-responses (including the highest concentrations), but we do not present these models because the third-order fits often unrealistically exceeded 100% survival. Time to death could not be analyzed for *Laboratory Experiment II* because it was a missing cells design (i.e., for each species some levels of chlorothalonil had no mortality).

All main effects and interactions were significant for percent survival (Species: $F_{2,123}$=82.53, P<0.001; Chlorothalonil: $F_{6,123}$=57.45, P<0.001; Species x chlorothalonil: $F_{12,123}$=5.20, P<0.001; Figure 2C, see Figure S1 for mortality through time). For *R. sphenocephala*, 0.164µg/L caused significantly more mortality than each adjacent concentration and there was a significant quadratic term for the dose response <97µg/L (log chlorothalonil: $F_{1,49}$=9.93, P=0.003, log chlorohtalonil^2: $F_{1,49}$=8.67, P=0.005). For *H. cinerea*, 0.0164µg/L caused significantly more mortality than each adjacent concentration and, like for *R. sphenocephala*, there was a significant quadratic term for the dose response (<16.4µg/L; log chlorothalonil: $F_{1,21}$=6.82, P=0.016, log chlorohtalonil^2: $F_{1,49}$=7.43, P=0.013).

Chlorothalonil concentration was associated quadratically with immune responses (Chlorothalonil: Wilk's $F_{2,43}$=9.84 P<0.001, Chlorothalonil^2: Wilk's $F_{2,43}$=14.20 P<0.001; Figure 3B). This quadratic relationship was observed for both melanomacrophages (Chlorothalonil: $F_{1,40}$=14.09 P<0.001, Chlorothalonil^2: $F_{1,40}$=18.75 P<0.001) and granulocytes (Chlorothalonil: $F_{1,40}$=10.38 P=0.002, Chlorothalonil^2: $F_{1,40}$=16.66 P<0.001; Figure 3B)

Corticosterone Experiment: Methods

Tadpoles were obtained by pouring the water from each jar through a net, and the tadpoles were immediately transferred to a beaker of 10% benzocaine sitting in dry ice. This immediately euthanatized and flash froze the animals with a handling time of typically less than 10 seconds, which is not expected to affect corticosterone levels. Each homogenized tadpole (homogenized with a Power Gen 125 homogenizer, Fisher Scientific) was extracted twice with a 7:3 ethyl ether - petroleum ether cocktail (4ml per 1ml homogenate). The supernatant was removed from the water fraction by freezing the water and decanting the ether cocktail, which was collected in a 13x100mm glass vial and dried under a pure nitrogen stream at 40°C. The purpose of the petroleum ether was to help separate the fats into a monolayer so we could effectively collect the steroid layer, and the freezing cycle helped to precipitate out excess fat and other unwanted compounds.

A corticosterone EIA kit was then used to quantify hormone levels in each sample (Assay Designs: cat# 900-097, Ann Arbor, Michigan). Immediately before running the assay, dried samples were reconstituted in 500 µL of Assay Buffer 15, mixed with 12.8 µL of steroid displacement buffer, and vortexed vigorously. This reconstituted sample extract (50 µl) was then added to wells (along with enzyme conjugate and other reagents) and incubated for 1h, during which the corticosterone binds to the antibody attached to the plate well. Next the plate was washed three times with washing buffer, leaving an empty and colorless well prior to the addition of reactive reagents to complete the assay. Plates were read using a 48-well ELISA plate reader (Bio-Tek, Winooski, VT). Samples were run in duplicate following the manufacturer's instructions for the EIA kit, and standard curves for each run of the assay (n = 2) spanned a 20,000-32 pg range. Inter-assay variation was 11.6%, and intra-assay variation was 7.6%.

We added 250µCi of tritiated corticosterone to each sample prior to the extraction step, to allow us to determine the efficacy of the extraction protocol. Following the extraction and reconstitution steps, we used a scintillation counter to measure the amount of "hot" steroid remaining in 100 µL of each reconstituted sample. We then compared this value to a reading

from a non-extracted reference sample (i.e. 2000 cpm) to determine the percent of corticosterone that was lost or bound by lipids during the extraction process. Recoveries varied among individual tadpoles, as expected due to large among-tadpole variations in lipids, but were consistent among replicates of the same homogenates during pilot studies.

It is important to recognize that extractions from tadpole homogenates contain some compounds in addition to corticosterone that might influence ELISA assay results. These might include compounds that cross-react with the anti-corticosterone antibodies, or compounds that inhibit corticosterone binding to the antibodies. Cross-reactivity was probably minor, given that the only steroid other than corticosterone with more than 2% cross-reactivity is deoxycorticosterone (28.6% cross-reactivity), which is also released during a typical stress response but at low levels relative to other stress hormones (Reddy 2006). The possible presence of inhibitors, which might include lipids and other compounds, is more difficult to assess and might vary according to tadpole size and stage. However, tadpoles were of similar stages in this experiment in addition to being randomly assigned to treatments, so inhibitors were almost certainly randomly distributed across treatments. Therefore, any inhibitors present in the extracted samples should increase random variation in the assay, making it more difficult to detect treatment effects in the experiment. Hence, if inhibitors were present, they should have increased the chances of a false negative, not a false positive, result.

Reddy, D. S. 2006. Physiological role of adrenal deoxycorticosterone-derived neuroactive steroids in stress-sensitive conditions. Neuroscience **138**:911-920.

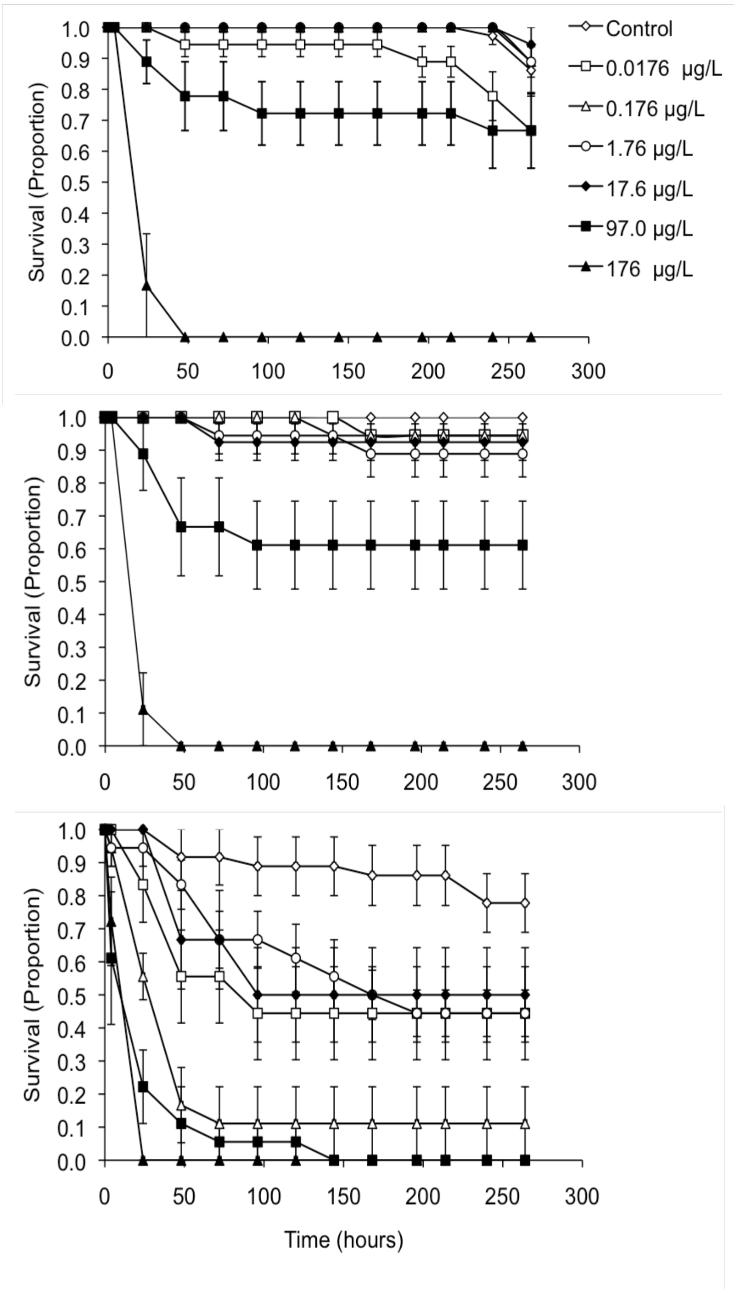

Figure S1. Survival of tadpoles in *Laboratory Experiment II* through time. Survival of *Hyla cinerea* (A), *Osteopilus septentrionalis* (B), and *Rana sphenocephala* (C) exposed to several concentrations of chlorothalonil (0.0164, 0.164, 1.64, 16.4, 82.0, and 164 µg/L) and controls (water and solvent presented together) for *Laboratory Experiment II*. Shown are the means (±SE).

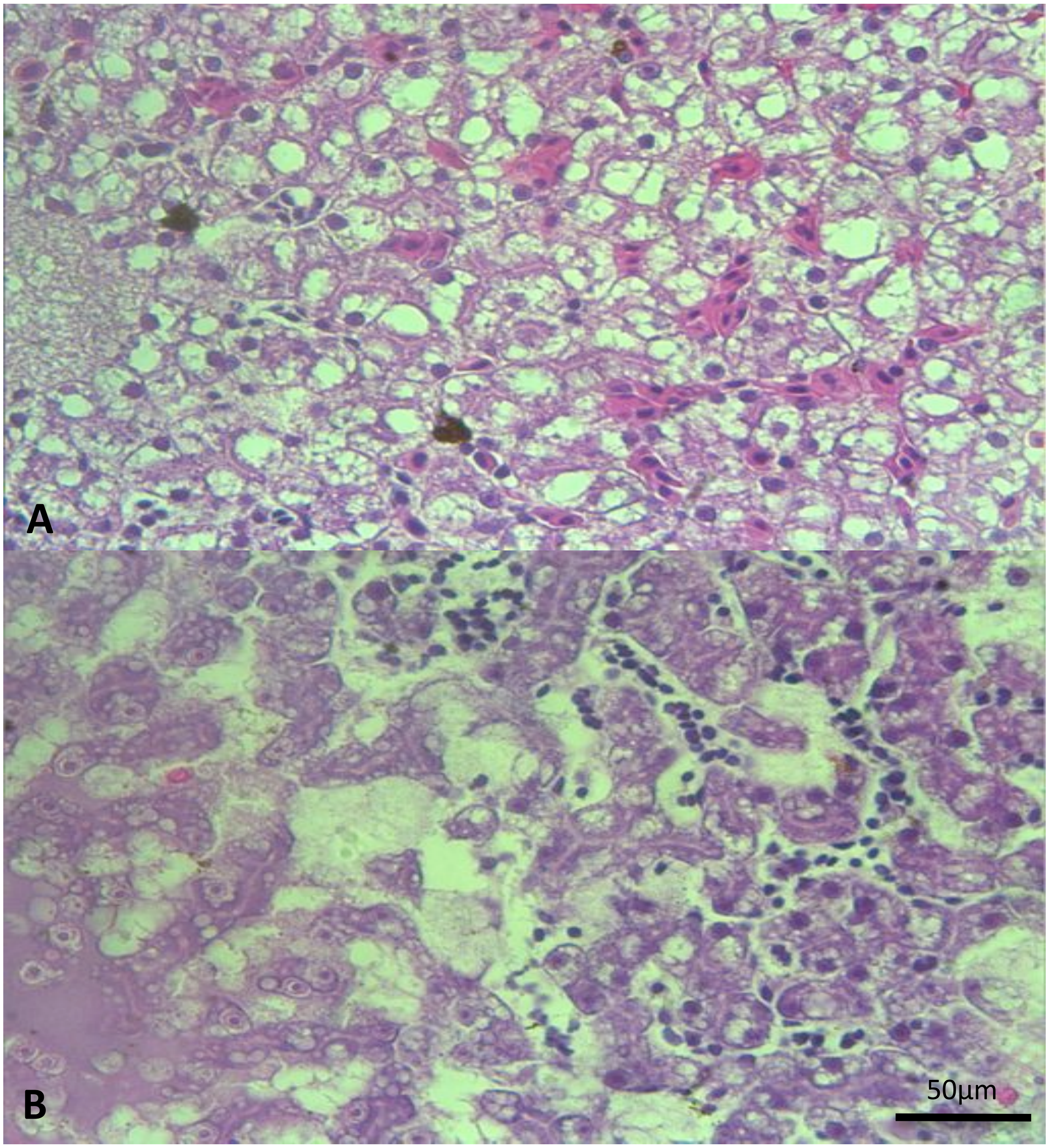

Figure S2. Representative photomicrographs of sectioned and stained livers of *Osteopilus septentrionalis* in control (A) and 164 µg/L chlorothalonil (B) treatments for *Laboratory Experiment II*. Note the difference in liver density between the photomicrographs.
